# Lightweight predicate extraction for patient-level cancer information and ontology development

**DOI:** 10.1186/s12911-017-0465-x

**Published:** 2017-07-05

**Authors:** Muhammad Amith, Hsing-Yi Song, Yaoyun Zhang, Hua Xu, Cui Tao

**Affiliations:** School of Biomedical Informatics, University of Texas Health Science Center, Fannin Street, Houston, USA

**Keywords:** Semi-automated ontology development, Public health, Natural language processing, Open information extraction, Ontology learning

## Abstract

**Background:**

Knowledge engineering for ontological knowledgebases is resource and time intensive. To alleviate these issues, especially for novices, automated tools from the natural language domain can assist in the development process of ontologies. We focus towards the development of ontologies for the public health domain and use patient-centric sources from MedlinePlus related to HPV-causing cancers.

**Methods:**

This paper demonstrates the use of a lightweight open information extraction (OIE) tool to derive accurate knowledge triples that can lead to the seeding of an ontological knowledgebase. We developed a custom application, which interfaced with an information extraction software library, to help facilitate the tasks towards producing knowledge triples from textual sources.

**Results:**

The results of our efforts generated accurate extractions ranging from 80–89% precision. These triples can later be transformed to OWL/RDF representation for our planned ontological knowledgebase.

**Conclusions:**

OIE delivers an effective and accessible method towards the development ontologies.

## Background

Ontology development, whether automatically generated or manually handcrafted, poses some specific challenges for success. Manually producing an ontology with an ontology editor such as Protégé [1], particularly with subject matter experts with very little or poor knowledge of ontology engineering, poses difficulty and confusion [2], and can be time consuming and resource intensive [3, 4]. Automated generation of ontology, known as ontology learning, from a body of corpora or data that contains pertinent knowledge for the ontology has yet to be perfected to produce satisfying results. A hybrid approach where manual development is augmented by automating some of the process would be a more feasible option to ease the initial development for ontology development. With human assistance, this would ensure that the development process is accurate, and advance any new knowledge on how to fully automate the workflow and technology based on lessons learned. This paper will introduce the use of open information extraction (OIE) to assist in the initial phase of extracting and filtering meaningful knowledge from resources, specifically patient-level cancer information from MedlinePlus [5]. We posit that open information extraction will elicit accurate extraction of knowledge tuples from patient-level textual sources for ontology engineering.

The term “Semantic Web” [6], coined by Sir Tim Berners-Lee, is a web of linked data, unlike the siloed information infrastructure of the current World Wide Web. The semantic web vision aims to integrate heterogeneous information sources and provide meaning through the use of ontologies that offer formal structural and symbolic representation of knowledge with annotations and vocabularies. With the use of ontologies (encoded in OWL/RDF format), not only one can map and merge a variety of distributed data sources, but also leverage the reasoning capabilities to provide inferences not explicitly found in the information. These benefits are of use in many domains, including the biomedical field, where there is continued effort to build and maintain ontologies for biomedicine and clinical decision support [7]. Some significant examples in this field include [8–13], and furthermore, the existence of the Semantic Web Health Care and Life Sciences Interest Group (HCLSIG), a W3C working group, promoting the use of semantic technologies to impact areas such as clinical and translational medicine, life sciences, and health care [14]. To a large extent, ontologies have and will have an important role in the discovery of new biomedical information and developing new technologies to assist clinicians and researchers.

**Fig. 1:**
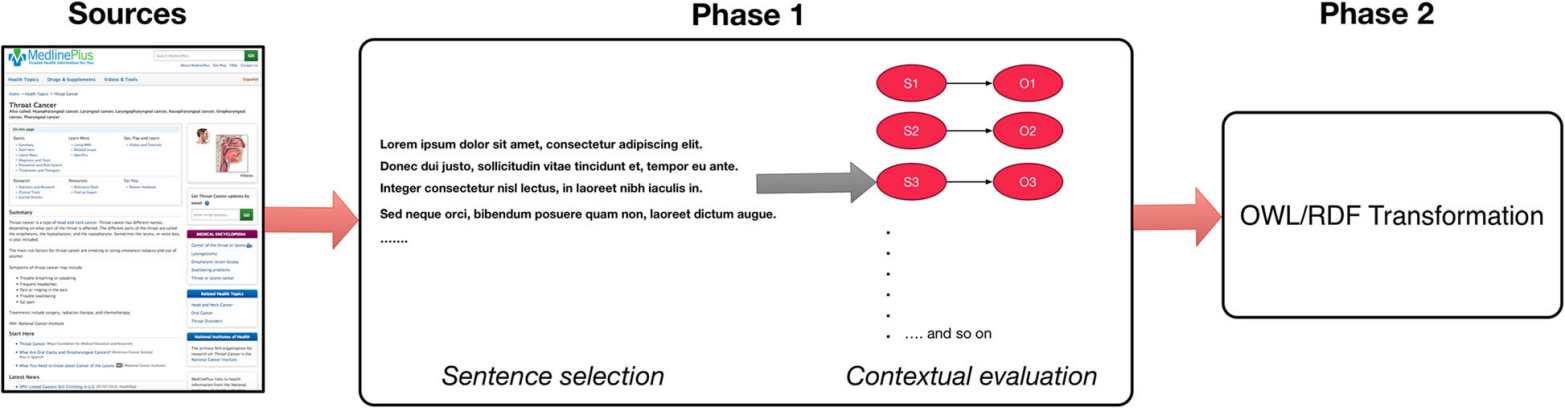
Transforming patient information to ontological format

Our overall outcome, which spans outside of the content of this paper, is to construct an ontology representing patient-level health information on HPV-related cancers. By leveraging the ontology, our ultimate goal is an interactive mobile assistant on patient-level HPV vaccine information to improve HPV vaccine coverage. A patient-centered ontology about HPV-related cancers will be an essential complement to a comprehensive knowledgebase of patient-level information on HPV vaccines. Figure 1 describes the totality of the process, with a focus on Phase 1 which this paper will cover^1^. Existing biomedical ontologies are tailored for professional biomedical professionals, and patient-level biomedical ontologies could bridge the knowledge and information between experts and patients, where an acknowledged gap exist. The linking of knowledge between the two populations could improve knowledge transfer to patients and health consumers. In addition, as expert biomedical ontologies have led to various tools and improved analytical processes, consumer biomedical ontologies could produce similar directions and tools for text mining and information retrieval. Helping to automate the process of ontology engineering can introduce new ontology authoring opportunities to individuals who would least likely to design and develop ontologies.

In the next section, we will discuss research of natural language processing for ontology engineering and some applicable research in the public health informatics domain. According to researchers, automating the creation of an ontology is a near impossible feat to accomplish as evident with the diverse research on ontology learning in the biomedical or non-biomedical domains [3, 15]. However, there may be promise towards an semi-automated approach for constructing an ontology where some of the tasks or workflow could be facilitated and/or automated from conception to implementation [3, 15]. One natural language tool that has been employed in several studies is information extraction, where tuples of atomic, singular knowledge is extracted from textual sources.

### Open information extraction

Information extraction is a sub-field of natural language processing (NLP) that aims to retrieve sets of terms with relational information that link them. Often, the information retrieved is sets of entities bound by a relation [16]. Information presented in this format is useful for many application (mining biomedical text, ontology learning, and question answering), but within the context of this paper, it is of most use in ontology research. Ontologies structures knowledge as a set of terms with edges between them that are labeled as relational information to evoke meaningful information. Ontologies serve as the backbone of the semantic web concept which aims to provide meaningful information on the web [6]. Where ontologies could benefit from information extraction is in the development or population of ontologies (also known as ontology learning and population [17]), which this paper will elaborate and demonstrate.

Banko defines open information extraction as “a novel extraction paradigm that facilitates domain-independent discovery of relations extracted from text and readily scales to the diversity and size of the Web corpus” [18]. We paraphrase [19]’s excellent summarization on state of the art open information extraction in this paragraph. The distinguishing features of open information extraction, a sub-type of information extraction, is its domain-independence, its unsupervised methods to retrieve relational data, and scalability to large corpora. The first documented approach was TextRunner [18], which did not require a training set compared to previous approaches (Dipre [20], Snowball [21], KnowItAll [22]), employing a combination of dependency parsing, labeling of terms, and Naive Bayes probability values to each identified relation. Other OIE approaches emerged like Reverb [23]. Open language learning for information extraction (OLLIE) [24] is another important open information extraction method that utilizes dependency parsing with the Malt Parser [25]. It improves the resulting extraction triples by incorporating contextual information implied in the sentence and extracts additional triples by utilizing nouns and adjectives instead of solely relying verb-based predication [24]. Additionally, open information extraction systems, like OLLIE [24] or Reverb, either utilizes shallow parsing (parts of speech identification and chunking) techniques or very involved natural language processing methods, like dependency parsing. With the former, there is a cost of diminished recall for high precision, and the latter provides better precision and recall but with lowered efficiency [19].

The ClausIE Java-based library [19] developed by Max Planck Institute for Informatics is an unsupervised open information extraction module that produces triples (propositions) based on grammatical structure of the sentences. ClausIE takes a clause-based approach, identifying coherent pieces of information from free text, to produce predicates. ClausIE relies on the Stanford Parser’s dependency parser [26] and decision rules to detect clauses, and their approach is noted to be more accurate than related open information extraction systems (like TextRunner and Reverb). Most open information extraction utilize rules to parse out triples based on a dependency tree. ClausIE does the same in detecting clauses from the dependency tree, and then based on the type of clause it formulates the proper subject-predicate-object information from grammatical patterns in the English language. In addition, ClausIE permits users to configure the output of the results, so options like representing the tuples as n-ary format, or expanded knowledge decomposition are available.

### Related studies

The body of research on ontology learning, and semi-/complete automatic development of biomedical ontologies are numerous, according to a thorough survey by [15]. The phases of development of ontology is divided into seven tasks, the discovery of 1) terms, 2) synonyms, 3) concepts, 4) hierarchal concepts, 5) relations, 6) hierarchal relations, and 7) axioms [17]. This paper focuses on relational extraction, which defines the links between concepts and entities in an ontology to evoke meaningful knowledge. By discovering the relational links, we may be able to ascertain the terms and concepts in a future step. Precisely, relational terms are not only the explicit information derived, but also the implicit information that describe “is a” relations (See “ClausIE library” section for an example). Intuitively, information extraction methods that can produce explicit and implicit relational information can be a beneficial tool for ontology engineering to derive every piece information to produce a comprehensive ontology. Our intended focus is the development of health-related ontology, and to preform the development process in an easier and relatively faster way for the non-ontologist domain experts.

**Fig. 2:**
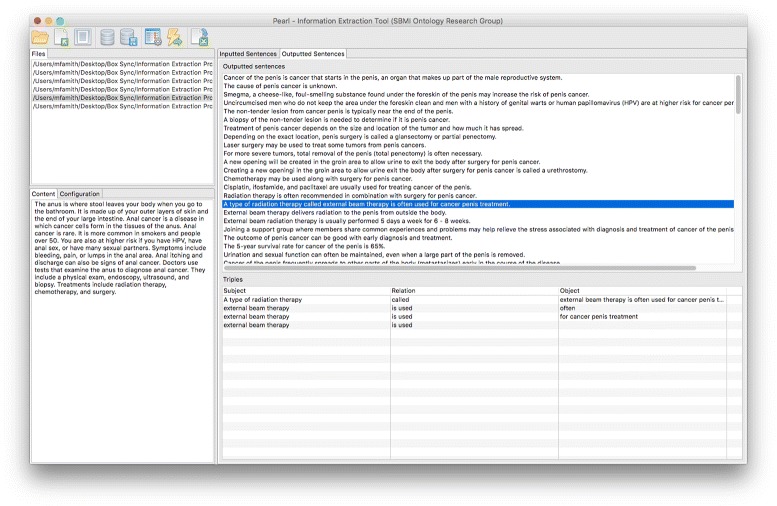
Pearl Information Extraction Kitchen (PIE KIT)

SemRep, a rule-based NLP tool supported by the National Institute of Medicine, identifies semantic predications as defined in the semantic network of UMLS [27]. Rosemblat, et al. aimed to build an ontology in public health promotion to support the extension of SemRep [28]. Our approach uses an open information extraction NLP tool, which gave us the flexibility to develop a customized ontology representing the conceptualization of the specific domain. In contrast, SemRep harnesses the use of existing terminological hierarchy and semantic relationships contained in the existing ontology of UMLS, to derive semantic predications constraint in the semantic network. Besides, SemRep is only supported by Linux, whereas open information extraction tools like ClausIE are lightweight and platform independent. We developed an in-house tool with convenient user interface on top of ClausIE, which could be deployable in Windows, MacOSX, and Linux (Fig. 2). Within the biomedical domain, other workflow-regulated ontology development tools have been introduced like [29], but do not utilize natural language methods for automation.

### Our contributions

This paper offers several potential contributions. Firstly, we introduce the use of open information extraction, specifically ClausIE, from a corpus of patient health information related to cancer. Similar in purpose as SemRep, ClausIE is a lightweight information extraction library that produces knowledge triples in subject, predicates, objects or n-ary representations. Secondly, this study offers a semi-formal method to assist subject matter experts to initialize the development of an ontology from textual sources, and a front-end user tool that can enable novice subject matter experts to easily utilize ClausIE. Lastly, this study also proposes a formalized evaluation criteria to guide subject matter experts to assess the results. In the next few sections, we discuss the process and tools utilized for this study, and discuss the results from the process and outline limitations and future direction for the next phase.

Thusly, we intend to find out if extracted knowledge of patient health information is optimal for ontology serialization with ClausIE. ClausIE provides various output options to enhance the output representation. Therefore, we need to determine an appropriate extraction configuration within ClausIE that will provide an accurate export of triples to help seed the development of an ontology for patient-level knowledge of cancer information. We aim to discover if a specific extraction of propositions contribute to accurate tuple information with the original source, and precise decomposition of the tuples. Additionally with decomposition, we also aim to learn if n-ary representations provided by ClausIE can improve the decomposition of the tuples without impediments.

## Methods

### MedlinePlus corpus

In an effort towards building a knowledgebase for consumers, MedlinePlus has been chosen for the friendly language, reliability, and coverage of the health information provided. MedlinePlus is a website produced by the National Institutes of Health (NIH) that provides health information curated for patients, their families and friends, and other consumers [30]. The health information covered by MedlinePlus is updated daily based on information from NIH and other trusted sources. The content ranges from description of diseases, meanings of drugs, to videos and links to the latest treatment and other relevant news. By the year of 2015, 975 health topics have been provided by MedlinePlus [31]. Web pages from the categories of “Health Topics” and “Medical Encyclopedia” of MedlinePlus were selected in this study due to their relevance and comprehensiveness. A typical web page of a disease from either “Health Topics” or “Medical Encyclopedia” includes sections highlighting the definition, causes, symptoms, treatments, preventions, and other aspects of a disease. Therefore, MedlinePlus has been chosen as an ideal source for sample sentences on cancer information at the patient-level.

The web pages about HPV-related cancers from the categories of “Health Topics” and “Medical Encyclopedia” of MedlinePlus were parsed and the text content was saved as plain-text files for later knowledge triple extraction in this study. For this, we first retrieved the HPV-related cancers based on the HPV Vaccine Information Statement provided by the Centers for Disease Control. Six HPV-related cancers were included: anal cancer [32], cervical cancer [33], penis cancer [34], throat cancer [35], vaginal cancer [36], and vulvar cancer [37]. Documents regarding HPV-related cancers were then gathered from MedlinePlus. Six documents were selected from MedlinePlus as the start of this project, with one document introducing one HPV-related cancer. More relevant documents could presumably be included in the future if necessary.

### ClausIE library

Mentioned before, this study utilizes the open information extraction library, ClausIE, to derive knowledge tuples for ontology engineering. This NLP library is ideal for this study for several reasons. One in particular is that the library is reliant primarily on dependency parser and grammatical sentence structure to evoke knowledge triples, as opposed to large, “heavy-weight” approaches like SemRep^2^. Not only can explicit knowledge triples be derived from this method, but also implied, embedded knowledge can also be evoked. Take for example, a sentence like “The human papillomavirus virus (HPV) leads to cervical cancer” would produce an explicit triple (“The human papillomavirus virus”, “leads to”, “cervical cancer”) and an implicit triple (“human papillomavirus virus”, “is”, “HPV”). In addition, ClausIE is domain independent and when rated against other well-known domain-independent, open information extraction approaches, performance is significantly better [19].

Further details of ClausIE’s method can be found at [19], but essentially, ClausIE relies on a dependency parser to syntactically analyze a sentence. Based on the results of the dependency parser, clauses, which are coherent pieces of information, are determined by ClausIE. Then, using ClausIE’s decision tree rules and grammar-based rules, the system identifies the type of clauses, and from the types of clauses, propositions/triples are generated.

### Configuration

We intended to create several datasets based on various configuration to retrieve the optimal output appropriate with respect to accurate knowledge triples for ontology development. Three datasets were created based on three configurations respectively.

The (1) utilized the default ClausIE settings (“Default”). The clause detection of “Default” included 1) extracting propositions from principal modifiers (Process Modifiers from Table 1), 2) assuming subject-verb-adverbials (SVA) clauses where sentence does not contain a complement or an object (Conservative SVA from Table 1), and 3) detecting clauses from coordinating conjunction verbs (Conjugate Verbs from Table 1).

**Table 1:** Dataset options for extraction

Representation	Default	+SVOA	+SVOA_NVERB	Default (n-ary)	+SVOA (n-ary)	+SVOA_NVERB (n-ary)
n-ary	No	No	No	Yes	Yes	Yes
Clause detection						
Process modifiers	Yes	Yes	Yes	Yes	Yes	Yes
Conservative SVOA	No	Yes	Yes	No	Yes	Yes
Conservative SVA	Yes	Yes	Yes	Yes	Yes	Yes
Conjugate verbs	Yes	Yes	Yes	Yes	Yes	Yes
Conjugate non-verbs	No	No	Yes	No	No	Yes

The (2) includes the “Default” settings and the addition of clause detection rule for recognizing adverbials to distinguish subject-verb-object (SVO) and subject-verb-adverbial (SVOA) in ClausIE detection rules (Conservative SVOA from Table 1). This configuration will be referred as “Default+SVOA” in the following sections.

The (3) incorporates the options from “Default+SVOA” and the detection of clauses based on non-verb coordinating conjunctions (NVERB CC). This configuration will be referred as “Default+SVOA+NVERB CC” in the following sections.

Also by default, the extracted data is presented as triples, namely tuples of subject, verb, and object. An option is available to extract the data as n-ary tuples where there are one or more arguments. In addition to processing the data based on the options described before, this study will evaluate data in both triples and n-ary tuples, a total of six datasets.

### Sentence selection and modification

**Fig. 3:**
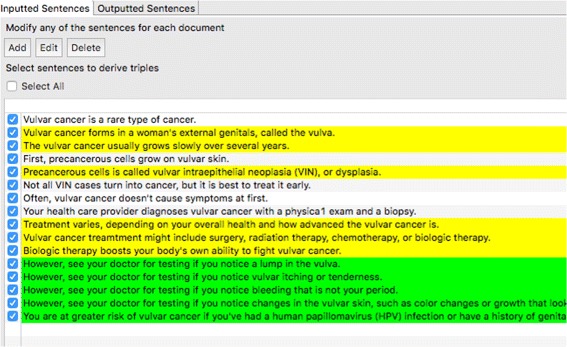
Screenshot of the sentence selection

The sentence selection and modification process was conducted manually with the help of the interface “Pearl” Information Extraction Kitchen (PIE KIT) developed in this study (Fig. 3). More description of PIE KIT is available in the results section. Each sentence was examined and possibly edited before it was processed by ClausIE to be used for the ontology we plan to design.

As for sentence selection, we only included sentences with information useful for the ontology. This assumes that the information has data that could be added to the ontology based on the ontology’s purpose. If the information in the sentence is not useful, the sentence is not selected since it has no potential value to the ontology.

As for sentence modification, we have either edited the sentences (e.g. add, replace, or remove words) or divided one sentence into several sentences by adding new sentences and revising the original sentences. For specific examples of sentence modification, please check “5.1 Sentence selection and modification” in the discussion section. The criteria used for sentence modification were (1) grammatically proper format and (2) understandability of a sentence without context. These two rules are explained below:

1. *Grammatically proper format.* It is essential to have the selection formatted as a grammatically proper sentence. Some information imported as a sentence may be a subtitle or header appended to the sentence, or sometimes bullet list content is treated a sentence. In these cases, if encountered, the sentence is edited or divided into separate new sentences by the PIE KIT users.
2. *Understandability of a sentence without context.* Neglecting the sentences before and after, the sentence should be coherent and understandable without contextual help. For example, sentences that may say, “This procedure is called a urethrostomy” [34] where “This” needs to modified to the exact name or description of the procedure. This criterion is to compensate for the lack of coreference resolution, which we will discuss in the future direction section.

### Contextual correct extraction evaluation

After processing with PIE KIT, and then exporting to CSV file, we evaluated the results for extraction correctness based on the context of the sentence and source from where it originated. Two individuals examined each triple from the datasets using an evaluation criteria, inspired by other information extraction studies [18, 19, 38] for contextual correctness. A third evaluator helped mediate disagreement of the extraction accuracy.

We extend on the discussion of our evaluation criterion with the following definitions:

#### **Definition 2.1**

(Tuple) In the context of this study, a tuple *t* is defined as a segment of words *w* that can either be a subject *s*, predicate *p*, object *o*, or an argument *a* (for n-ary representations).

$$ \forall \,[t] :=\{s,p,o,a\}, \text{where}\; \overline{t} := \left\lbrace w_{1} w_{2} \dots w_{n} \right\rbrace $$

#### **Definition 2.1.1**

(Binary Tuple) A binary tuple contains a sequence of ordered tuples that is a subject s and predicate p.

$$ \forall \ T_{2} \ \exists! \ s \wedge \ \exists! \ p $$

#### **Definition 2.1.2**

(Triple) A triple *T*_3_ is a sequence of tuples that contains one subject, one predicate, and one object.

$$ \forall \ T_{3} \ \exists! \ s \wedge \ \exists! \ p \wedge \ \exists! \ o $$

#### **Definition 2.1.3**

(Arguments) An argument set *AR* is defined as a set of tuples *t* of type argument *a* that is neither the subject or predicate for n-ary representations.

$$ \forall \ \overline{AR} := \left\lbrace a_{1}a_{2} \dots a_{n} \right\rbrace $$

#### **Definition 2.1.4**

(n-ary Tuple) An n-ary tuple is a type of tuple that contains one subject, one predicate, and a set of arguments.

$$ \forall \ T_{nary} \ \exists! \ s \ \wedge \ \exists! \ p \ \wedge \ \exists! \ AR $$

#### **Definition 2.2**

(Completeness) Completeness is a measurement to determine coherent thought from data. Completeness is determined by an existence of an object *o* or a set of arguments *AR* (for n-ary tuples). Binary tuples are excluded from this measurement.

$$ C = \left\{\begin{array}{l} \forall \ T_{3}, \ o \neq \varnothing \\ \forall \ T_{nary}, \ AR \neq \varnothing \\ \end{array}\right. $$

#### **Definition 2.3**

(Readability) Readability *R* is a measurement to signify if the data, assuming completeness, contains the subject *s*, predicate *p* and object *o* in a set of a specific order. With n-ary tuples, the order is subject *s*, predicate *p*, and with an argument *a*_*n*_ from the set of arguments *AR*.

$$ R = \left\{\begin{array}{l} \forall \ T_{2} := (s,p)\\ \forall \ T_{3} := (s,p,o)\\ \forall \ T_{nary} := (s,p,a_{n})\\ \end{array}\right. $$

#### **Definition 2.4**

*(Accuracy)* Accuracy *A* is denoted if data’s knowledge is reflected in the sentence *S* from which it originates.

$$A = \left\{\begin{array}{l} \forall \ T_{2} \in S \\ \forall \ T_{3} \in S \\ \forall \ T_{nary} \in S \end{array}\right. $$

#### **Definition 2.5**

(Connectivity) Specific for n-ary tuples, connectivity ∼ relates that every arguments *a*_*x*_ must modify a tuple *t*_*x*_, either the subject *s*, predicate *p*, or another argument $ a_{x_{n \neq n}} $.

$$ \begin{aligned} CT &= \left\{ \forall \ T_{nary} \in \ \exists \ a_{x} \sim t_{x} \colon \ a_{x}=\left\lbrace a_{x_{1}}, a_{x_{2}}, \dots, a_{x_{n}} \right\rbrace\right.\\ &\left.and~t_{x} = \left\lbrace s, p, a_{x_{n \neq n}}\right\rbrace \right\} \end{aligned} $$

As for binary tuples, the contextual correctness includes readability (2.3), and accuracy (2.4). As for triples, the contextual correctness includes completeness (2.2), readability (2.3), and accuracy (2.4). As for n-ary tuples, contextual correctness includes completeness (2.2), readability (2.3), accuracy (2.4), and connectivity (2.5).

N-ary tuples require different criteria due to the complexity of the information provided. The addition of connectivity (2.5) involves examining all arguments and assessing their relationships with other arguments, subject, or predicate. If each argument is extracted correctly to either act as the object or the modifier of the subject, predicate, or other arguments, it is considered contextually correct.

## Results

For usability purposes in service of novice technology users, we developed a front-end Java application on the Eclipse client platform, called “Pearl” Information Extraction Kitchen (PIE KIT). PIE KIT interfaces with the ClausIE, and it is a tool that allows users to import plain-text documents from a folder location or individual files, and then automatically parses the sentences from the body of text. PIE KIT enables users to make edits to individual sentences, or ignore sentences, for subsequent processing in obtaining individual pieces of knowledge. Also, it permits the user to configure ClausIE, and it also provides a feature to save the project files for later retrieval and processing. After processing the sentences, the tool displays the results and export to a CSV file format. At a later stage, we ideally would like to integrate additional tools and libraries to make it a comprehensive workbench for a guided and semi-automated system for ontology development. Additional enhancements will be discussed in the future direction section.

To develop a cancer-related ontology for patients, we have sampled 107 sentences from 6 documents on HPV-related cancers based on information provided by MedlinePlus. We then manually selected 106 sentences from the total 107 sentences. Among the 106 selected sentences, 29 were manually created and 45 were modified before processing by ClausIE. It was in response to either poor sentence detection or to elaborate on missing contextual information. For the entire corpus, we produced 6 outputted datasets of triples based on the options described in Table 1. The main difference between half of the output was the n-ary configuration that allowed for n-ary tuples instead of the standard triples. The *Default+SVOA*for triples and n-ary included not only the default configuration but also the identification of SVOA clause patterns. *Default+SVOA_NVERB* carries over the aforementioned settings, and processing and detection of conjugate non-verbs for decomposition of information to atomic pieces.

As a result of the processing, the Default setting dataset yielded a total of 303 unique triples. Both *Default+SVOA* and *Default+SVOA+NVERB_CC* produced 262 and 345, respectively. Table 2 presents the accuracy of the extraction based on the criteria described in “Contextual correct extraction evaluation” section. *Default+SVOA* exhibited the highest accuracy from the other two, yet minimally better (85.1%). *Default* and *Default+SVOA+NVERB_CC* both had 80.2 and 83.5%.

**Table 2:** Contextual accuracy results for outputted triples

	Correct	Incorrect	Accuracy (%)
Default	243	60	80.2
Default+SVOA	223	39	85.1
Default+SVOA+NVERB_CC	288	57	83.5

Table 3 presents results from the processing with n-ary output. Both the *Default* and *Default+SVOA* produced the same number total number of tuples, 191, and same number of correct and incorrect tuples, 170 and 21. Additionally, the accuracy, based on the n-ary contextual evaluation criteria described in “Contextual correct extraction evaluation” section, with 89.0%. The *Default+SVOA+NVERB_CC* provided 231 correct tuples and 28 incorrect tuples (259 total number of tuples) with an 89.2% accuracy.

**Table 3:** Contextual accuracy results for outputted n-ary tuples

	Correct	Incorrect	Accuracy (%)
Default	170	21	89.0
Default+SVOA	170	21	89.0
Default+SVOA+NVERB_CC	231	28	89.2

In the following section, we will elaborate on our results and assume the appropriate extraction possibilities using open information extraction for ontology development, including a summarization of sentence-level correctness among the 6 information extraction options for further examination.

## Discussion

### Sentence selection and modification

Noted earlier, 106 out of 107 sentences were chosen for information extraction. The one sentence excluded was “Doctors prescribed DES in the 1950’s to prevent miscarriages” due irrelevancy for the ontological knowledgebase to be developed.

Forty-five out of the 106 were modified due to provide some contextual information. For example, the sentence from the Vaginal Cancer document [36] contained “If your results are abnormal, you may need a biopsy or other tests” was changed to “If your Pap test results are abnormal, you may need a biopsy or other tests”, adding “Pap test” to frame “results”. Another example, “See: Cancer - support group Outlook (Prognosis) The outcome can be good with early diagnosis and treatment” [34] was edited because of malformed imported sentences that included unnecessary header information. This was later modified to “The outcome of penis cancer can be good with early diagnosis and treatment”.

Twenty-nine sentences had to be created mostly to deal with list-like passages that were bulleted or malformed due to poor sentence detection. From the document for throat cancer [35], “Throat Cancer Also called: Hypopharyngeal cancer, Laryngeal cancer, Laryngopharyngeal cancer, Nasopharyngeal cancer, Oropharyngeal cancer, Pharyngeal cancer Summary Throat cancer is a type of head and neck cancer” was retrieved from the document. The imported passage led to the creation of 7 new sentences:

- Throat cancer also called Hypopharyngeal cancer.
- Throat cancer also called Laryngeal cancer.
- Throat cancer also called Laryngopharyngeal cancer.
- Throat cancer also called Nasopharyngeal cancer.
- Throat cancer also called Oropharyngeal cancer.
- Throat cancer also called Pharyngeal cancer.
- Throat cancer is a type of head and neck cancer.

Nearly half of the sentences used for the study (45%), required intervention from user to prepare the data for extraction. While the tool was able to import and parse out the data based on sentence needed for processing and providing an accessible mechanism for users to modify the data, investigating possibilities to improve sentence detection that accounts for headers and bullet points passages and to incorporate contextual relationship into the sentence would improve automation and save time for overall development.

### Comparing Default with Default+SVOA

We compared the results between Default and Default+SVOA. Data produced from former yielded 303 triples compared to the latter of 262, with a contextual accuray of 80.2 and 85.1% respectively. Default+SVOA removed what deemed to be redundant triples that existed in Default’s dataset. While most of the extracted data were similar for the sentences, 34 instances among the Default+SVOA dataset deviated from the Default with redundancies. An example would be the sentence “Anal cancer symptoms include bleeding, pain, or lumps in the anal area” [32] which yielded the following triples for the Default dataset:

1. (“Anal cancer symptoms”, “include”, “bleeding pain or lumps in the anal area”)
2. (“Anal cancer symptoms”, “include”, “bleeding pain or lumps”)

Yet, Default+SVOA only elicited only the first triple from above. In one instance, the redundancy did cause information loss for the sentence, “A new opening will be created in the groin area to allow urine to exit the body after surgery for penis cancer” [34]. From a pragmatic perspective, the addition of SVOA detection removed unnecessary knowledge triples that contributed to inaccuracies, thereby improving the accuracy for Default+SVOA dataset.

### Comparing Default+SVOA with Default+SVOA+NVERB_CC

When comparing Default+SVOA with Default+SVOA+ NVERB_CC, there exist some deviation with the resulting data. Between the two datasets, 36 sentences differed in output due to decomposition to additional propositions. For example a sentence like, “Vagina cancer treatment might include surgery, radiation therapy, and chemotherapy” [36] produced the following triples for the Default+SVOA:

1. (“Vagina cancer treatment”, “might include”, “surgery radiation therapy and chemotherapy”)

With the same sentence, Default+SVOA+NVERB_CC produced the following triples:

1. (“Vagina cancer treatment”, “might include”, “surgery”)
2. (“Vagina cancer treatment”, “might include”, “radiation therapy”)
3. (“Vagina cancer treatment”, “might include”, “chemotherapy”)

In the example above, we attained a better decomposition of the data, which could inevitably help represent the information into RDF.

With better decomposition, the accuracy of information extracted was lower compared to the previous datasets discussed at 83.5%. In a few of the sentences, that noticeably had deeper decomposition, contextual errors were generated. In one sentence, “However, see your doctor for testing if you notice changes in the vulvar skin, such as color changes or growth that look like a wart or ulcer” [37] produced several erroneous triples:

1. (“color changes”, “look”, “like a wart”)
2. (“color changes”, “look”, “like a ulcer”)
3. (“color growth”, “look”, “like a wart”)
4. (“color growth”, “look”, “like a ulcer”)

The above sample of the dataset are all incorrect based on what is stated in sentence. Comparatively to the Default+SVOA result produced:

1. (“color changes or growth”, “look”, “like a wart or ulcer”)

While the above example was not decomposed, it is still contextually correct based on our guidelines.

### Comparing triple representation with n-ary representation

Another set of dataset mirrored the above discussed results but was outputted in n-ary tuples to assess better accuracy. Table 3 summarizes accuracy for each version, Default (89.0%), Default+SVOA (89.0%), and Default+SVOA+NVERB_CC (89.2%). Overall, compared to triple-based dataset (Table 2), the n-ary results exceeded accuracy of the previous. Despite the addition of n-ary extraction, both Default and Default+SVOA results were similar with no deviation or difference, hence the same contextual accuracy. However, the addition of non-verb coordinating conjunction option for ClausIE generated slightly better accuracy.

When comparing Default+SVOA+NVERB_CC to its non-nary counterpart, high contextual accuracy was due to malformed exported data that contributed to either poor readability, completeness, or accuracy. There was also redundant data that contributed to these issues. For example the sentence from the non-nary dataset, “The non-tender lesion from cancer penis is typically near the end of the penis” [34], produced the following two triples:

1. (“The non-tender lesion from cancer penis”, “is”, “typically near the end of the penis”)
2. (“The non-tender lesion from cancer penis”, “is”, “typically”)

The former was deemed correct by the evaluators, but the latter was denoted as an error and incorrect based on our criteria. Issues with adverbs (when, what, where, also, etc.) appended to the data’s object distorted the contextually accuracy. Overall, the n-ary representation evoked better decomposition of the information as arguments to make the propositions readable, complete and accurate.

Considering the high precision of one of the n-ary dataset, we examined the results for opportunities to improve the information extraction method. We determined four types of issues that may have resulted in some of errors in the dataset - interference by adverbs, erroneous clause detection, unsystematic decomposition of conjugates, and ambiguous contextual accuracy.

With interference by adverbs, certain triples or n-ary tuples included, what was deemed to be, unnecessary adverbs (when, where, what, etc.) as arguments. For example, the sentence, “Urination and sexual function can often be maintained, even when a large part of the penis is removed” [34], produced a n-ary tuple, (“a large part of the penis”, “is removed”, “when”). “When” was an argument that was produced and led to contextual inaccuracy when evaluated. Adverbials, perhaps due the complexity of its usage in sentences, presents some challenges or limitations for the ClausIE library [19]. However, ClausIE does offer a facility to create a stop-word list for adverbs to ignore while processing, and in all of the cases, removing the adverb argument would have resulted in contextually correct information.

Three of the errors were due to ambiguity of contextual accuracy. One example is the sentence “Doctors use tests that examine the anus to diagnose anal cancer” [32] where the extraction produced a proposition, (“the anus”, “to diagnose”, “anal cancer”). The evaluators agreed that proposition was incorrect since according to the sentence, it is the tests that are used to diagnose anal cancer. Yet, it is reasonable to assume that a bodily region may be used to indicate anal cancer.

One of the remarkable features of ClausIE is the ability to produce decomposed propositions from conjugates in the sentences. Despite this ability, a handful of errors resulted due to how the sentence was scripted. An example from [34] where it contained the sentence “Penis cancer treatment includes surgery that cuts and remove the cancer”, produced the following tuples:

1. (“Penis cancer treatment”, “includes”, “surgery”)
2. (“surgery”, “cuts”)
3. (“surgery”, “remove”, “the cancer”)

(2) was labeled as incorrect because ideally it should be similar to (3) and state (“surgery”, “cuts”, “the cancer”). One could make a case that (2) may be correct despite the incomplete coherency. When we edited the sentence to “Penis cancer treatment includes surgery that cuts the cancer and remove the cancer”, we did receive a corrected version of (2).

The ClauseIE is contingent on dependency parser to detect clauses, which would lead to the derivation of propositional tuples. Depending on how the sentence is organized the results of the tuples may vary, so certain uses of appositions and abbreviations could skew the dependency parsing based on our examination of the dependency parser output^3^. In some of the erroneous cases, an apposition was associated with a word beside it, resulting in a proposition like (“the penis”, “is”, “total penectomy”) from the sentence “For more severe tumors, total removal of the penis (total penectomy) is often necessary” [34]. Similar have occurred with abbreviations. Additionally, the parsing issues also emerged with complex construction of the sentences, sometimes as a consequence of the editing of data before processing. In other words, adding contextual information to a sentence like “This procedure is called a urethrostomy” to a more descriptive authored version “Creating a new opening in the groin area to allow urine exit the body after surgery for penis cancer is called a urethrostomy” produced different results. In some examples, an inspection of the parsing tree where a “dep” label would indicate that the parser is unable to discern the structure of the sentence, or it may have incorrectly identified root verb. In regards to the latter, from [37], initially the sentence was “It forms in a woman’s external genitals, called the vulva” but changed to “Vulvar cancer forms in a woman’s external genitals, called the vulva” where the dependency parser identified “called” as the root verb instead of “forms”. Some of these issues could be avoided by either investigating solutions for co-reference resolution or associating some meta-information manually by the user and keeping the sentence as is.

While 89.2% is a relatively high precision score for contextual accuracy, the rectification of some of the above-mentioned issues could produce some useful results for ontology engineering and other related text mining endeavors. Additional future opportunities will be discussed in the subsequent final section.

A spiritual predecessor to our study [28] demonstrated the use of SemRep [27] to extend an existing ontology to support public health related concepts. SemRep is a domain-specific information extraction that produced an extraction accuracy of 85%, yet with our study we produced an accuracy as high as 89.2%. Also, [28] was limited by domain dependency issues of SemRep, and lack of support for precise detection of morphological structures of sentences, according to the authors. These limitations were addressed with our study. Finally SemRep is a heavy-weight system that requires sophisticated knowledge and hardware to utilize, while PIE KIT aims to be a more lightweight and usable approach.

## Conclusions

The general objective of this paper is to assess the feasibility of utilizing open information extraction tools, specifically ClausIE, to aid in the retrieval of knowledge triples or n-ary tuples for ensuing encoding into an ontology. To evaluate the extracted knowledge triples, an evaluation criterion is needed to ensure contextual correctness and preservation of information.

Aside from applying the use of ClausIE for extracting knowledge intended for ontology development from patient-level documentation, this study introduced a tool to help facilitate the process of extracting knowledge through a desktop tool that harness ClausIE, PIE KIT. To formalize the evaluation of data for ontology engineering from information extraction tools, the study also provides some guidelines for selecting passages, and assess contextual correctness of extracted decomposed information.

The study explores the best extraction option with ClausIE that will provide knowledge tuples for OWL/RDF encoding. The results reveal that while enhancing the detection of clauses for knowledge triples with nonverb conjugation, the generated, desirable decomposed data came at the cost of accuracy, nonetheless, opting for n-ary decomposition helped improved the contextual accuracy. Moving forward, we realize that ClausIE would be a preferred tool for selecting and retrieving knowledge, and using PIE KIT while further developing and extending the software, could provide better facilitation of the knowledge engineering process for subject matter experts who lack the background knowledge for ontology development. The work presented is in an early phase toward the production of a consumer cancer ontology for patient-level information with some limitations to direct future research.

### Limitations

Our preliminary work included 107 sentences from a total of 6 documents of Medline webpages. Although the 107 sentences has represented patient-level information with different dimensions of HPV-related cancer information, it is relatively limited compared to the mass amount of information a patient or health consumer would have access to, such as social media and videos. We intend to further explore with larger number of sentences to further assess the feasibility of using open information extraction for ontology development, as well as experimenting with other subject matter relevant to patients, like vaccines.

### Future direction

Overall our next phase is to prepare the resulting data to be coded into a OWL/RDF format. Identifying terms and concepts is the next step. Theoretically by identifying the relational terms or potential labels for object and data properties, we can attain potential classes and instances based on the noun phrases and other parts of speech. We may utilize and integrate WordNet provide guidance for the user, and we may incorporate the work of [39] on their work relating to differential semantics to help distinguish parent classes and children classes. If possible it would be ideal to help the user by suggesting terms and similar terms to normalize or merge.

With PIE KIT, we allowed the user to edit the sentence if sentences were not properly extracted, and at times, the sub-headers were concatenated with the sentence or bulleted prose were viewed a one complete sentence. Since sentences were processed one at a time, another obstacle was contextual issues where pronouns referred to information previously described. In the future, improving the exact detection of the sentence is a future possibility to reduce the work for the user, and investigating possible solutions to handle contextual information surrounding sentences. Particularly, we are interested in co-reference resolution methods that could bypass the need for manually editing the sentences for context. As noted in the discussion, few of the errors could have been prevented without modifying the sentences.

Recently, Stanford NLP Group released an implementation of a clause-based information extraction that harness OLLIE [40]. Also, Bast and Haussman introduced Contextual Sentence Decomposition (CSD-IE) which is relatively comparable to ClauseIE but with a focus on better decomposition of information [38]. We also plan on experimenting with one of these recent to assess usability and performance, and if possible incorporate these new methods so that we can offer a more comprehensive platform to guide novice users to generate an ontology with PIE KIT.

Also to further standardize our evaluation criteria for extraction, we would like to present a methodology to assess extracted information’s transformation to OWL/RDF triples. This could help refine the selection of information to be encoded into an ontology and perhaps improve the evaluation presented in this paper.

## Endnotes

^1^ Phase 2 will be addressed in later research.

^2^ Standard release requires 71 GB. https://semrep.nlm.nih.gov/SemRep.v1.6_Installation.html.

^3^ ClauseIE utilizes the Stanford Dependency Parser.
